# Use of Mass Communication by Public Health Programs in Nonmetropolitan Regions

**DOI:** 10.5888/pcd16.190014

**Published:** 2019-07-25

**Authors:** Jennifer M. Kreslake, Allison Elkins, Christopher N. Thomas, Suzanne Gates, Thomas Lehman

**Affiliations:** 1 FHI 360, Social Marketing and Communication, Washington, District of Columbia; 2 Centers for Disease Control and Prevention, National Center for Chronic Disease Prevention and Health Promotion, Division of Nutrition, Physical Activity, and Obesity, Atlanta, Georgia

## Background

Mass communication is one component of effective public health program implementation (1). It includes news stories (“earned media”), paid media (advertising), and social and digital media (eg, social networking sites, text messaging, mobile applications, websites, blogs) (1). Earned media can increase the visibility of public health issues and support from community members and leaders (1). Sustained media campaigns are recommended population approaches to modifying diet, physical activity, and tobacco use behaviors (2). Mass communication using various channels has helped increase public awareness, knowledge, attitudes, and behaviors on a multitude of health topics (3,4).

From 2014 to 2017, the Centers for Disease Control and Prevention (CDC) funded 88 communities through Racial and Ethnic Approaches to Community Health (REACH) and Partnerships to Improve Community Health (PICH). REACH funded local health departments, tribes, universities, and community-based organizations to reduce racial and ethnic health disparities in their communities (5). PICH supported similar institutions to improve community health in cities, counties, and American Indian tribes or tribal organizations. Both focused on implementing evidence-based and practice-based strategies for tobacco use and exposure, physical activity, poor nutrition, and access to chronic disease prevention, risk reduction, and disease management opportunities (6). CDC asked funded programs to dedicate 10% of annual funding for mass communication. This was to support program objectives and share program messages, activities, and successes.

Many REACH/PICH programs had limited experience using mass communication to support public health efforts in rural or small towns. In response, CDC provided individualized training, guidance, and technical assistance. This included developing a communication plan, identifying and understanding key audiences, developing and pretesting messages and materials, selecting communication channels (eg, broadcast, print, outdoor, digital) and categories (eg, earned, paid, social or digital media), providing spokesperson training, conducting audience research, and evaluating communication activities.

To understand what contributed to successful communication efforts in nonmetropolitan regions, we conducted individual, 60-minute telephone interviews with personnel overseeing programmatic activities (“program managers”) and mass communication activities (“communication leads”) in 6 REACH/PICH programs. These programs achieved or exceeded annual communication objectives and dedicated at least 10% of annual funding to mass communication. Each interviewed program worked in municipalities with populations of 250,000 or less across multiple US Census regions. Two were tribal programs. By uing a semistructured interview guide, programs were asked open-ended questions about the challenges, opportunities, and promising strategies they encountered when implementing mass communication in small and mid-sized communities. Inductive qualitative analysis identified 4 emergent themes.

## Theme 1: Building Capacity for Mass Communication

For most interviewed programs, the perceived value and role of communication activities grew over time. Hiring communication staff or consulting services built long-term communication capacity. Increasing program capacity for mass communication had the potential to inform the organization more broadly, especially when staff who had gained communication training and experience advanced within the organization or worked across programs or grants.

Audience research helped programs develop communication materials for priority populations. Audience research and message testing helped programs define discrete audiences, better understand their messaging needs, and determine which channels were appropriate and accessible to audiences served.

Several programs felt prepared to continue their communication efforts after REACH/PICH funding because of the experience that had been gained. Programs also felt their communication efforts brought attention to the larger organization. Programs primarily conducted mass communication activities without direct involvement from their organization. CDC technical assistance and funding allocations provided support to help organizations understand and appreciate mass communication efforts.

### Quotes regarding capacity-building for mass communication

#### A budget allocation for communication conveyed its importance to program staff.

I think [the recommendations are] really important because it tells the awardee how important communication efforts are. . . . I think at the start of [the funding period] if you looked at [the program and] all the partners, there is no person who [was] designated to doing communication work. In a small community, that skill set and capacity wasn’t there, so what was really great about the [award] requiring that communication work, we put time and energy toward it to the point where now there’s a full-time person designated to work toward it. — Communication lead

#### Hiring communication staff or a consulting service built long-term communication capacity within the program.

It all depends on who you have on staff. . . . [Our communications person] knew a lot before coming here so she led the way with the communications without needing much hand-holding. — Program manager

Working with [the communication consulting firm] has massively increased our organizational capacity because we’ve learned so much about how formative research happens and about identifying what outlets we should be using, [and skills such as] plain language writing. . . . We’ve learned so much that we can use going forward about how to do our own messages. — Communication lead

[Hiring our communication lead] paid off tenfold. He created stuff and it was amazing. We needed that person. I think it should be [required] that you have a media person. — Program manager

Before PICH, we usually just relied on earned media and press releases. What would get picked up would get picked up, either for a news interview or an article in the newspaper. With the PICH funding, it has allowed us to have the staff time to put together our own commercials and then also pay for commercials to air on our local TV stations. — Program manager

#### Audience research helped programs define target audiences and understand their information needs.

I did communications plans with each of the program managers to get a little more detailed about who they needed to reach. . . . We had some nice audience worksheets that we were provided through the grant so I had worked through those, [and] message mapping. . . . They helped you really target in on identifying who those audiences were, what they did, what they looked like, in creating those nice personas. I think they work. I think they helped [program managers] say, “I get it. I don’t need to talk to everybody. I really need to talk to the people at the town who are doing this, or the human resources people at the businesses.” — Communication lead

One of the ways that we save money is that we did the focus groups ourselves. . . . Communications are very expensive, so making sure that we have the resources allocated to designing great messages that work with the community [was a consideration]. . . . We did a little backend testing with [materials], which actually was so helpful especially when making sure the language was appropriate. — Communication lead

We did a communication survey [in our community], which was great. We asked, “Where do you get your news from?” I think that’s something that every [program] should do. Because you have to know where your people get their information. I was surprised with our survey how many people still watch the news. — Program manager

I liked the materials we came up with. We worked really hard to get the messaging right, around not just healthy behaviors, [but] more around these environmental changes. . . . We spent a lot of money on that internal learning. — Communication lead

#### Programs had plans to maintain mass communication efforts.

The things we’ve learned through PICH, we will be able to apply to our ongoing communications with the public we serve [as a health department]. — Communication lead

Maybe a year and a half, almost 2 years into the project, all of the sudden . . . the light went on. We realized how sustainable communication objectives can be and how much of a difference just communicating a message can make in the community. . . . [The organization is] supportive, but I don’t think we had the knowledge as an organization. We didn’t do [message] testing of any sort. That was really eye-opening. We actually had a couple of staff . . . get trained in plain language writing. . . . We run everything through them now. — Communication lead

We have a visioning session and we do this once a year just to talk about what’s coming down. Even when REACH goes away, what’s next? Where do we go? How do we keep this momentum? We’ve been doing this for a couple of years and as part of this process we start every session with a SWOT analysis — the strengths, weaknesses, opportunities, the whole bit. It was interesting to me that yesterday, one of the things that was noted as a strength was communication . . . [and] it was also listed as an opportunity to get better. — Program manager

#### Programs felt their communication efforts brought attention to the larger organization.

The structure that this [REACH funding] was able to bring in regards to the amount of communication that had to be put out and the focus on a certain population made our organization realize, “Oh, we’re getting notoriety among partners and even community members.” . . . People knew, at the end of the day, that everything was linking back [to our organization]. — Communication lead

## Theme 2: Partners

Community-based organizations, local agencies, hospitals and educational systems, nonprofit entities, and local businesses may partner — formally or informally — to complement one another’s efforts. Programs worked with existing partners to increase the reach of communication efforts. Program partners were reliable and motivated to work with programs to promote messages and materials. Improving the communication capacity of partners was viewed by some as a method to achieve sustainability of program efforts.

Determining a core vision and aligning communication strategies advanced efforts of partner coalitions. Programs noted that many key partners for communication efforts were unpaid but shared similar goals. These partners frequently shared messages on behalf of the programs, expanding reach to audiences that may otherwise be less accessible.

### Quotes regarding partners

#### Unpaid program partners were reliable and motivated to work with programs to promote messages and materials.

We didn’t have too many paid partners. It was more of “grassroots for the betterment of the community” partner, or it was an initiative that directly benefited their audiences or their membership or their group. — Communication lead

#### Determining a core vision and aligning communication strategies advanced efforts of coalitions.

I was thinking . . . “How can we support our partners in getting a skill set that allows them to do their own communication work in the future?” Some of that is leading by example. Some of that is drawing partners into our work. For example, for making a video, making sure that partners who are interested in learning how to make videos are a part of that. We’d hold digital storytelling workshops, we’ve brought community partners to work with our media contractor. . . . That, to me, is another really strong way to sustain our effort. — Program manager

[Communication as a coalition] does require a conversation. The conversation needs to focus [on the fact that] this is not one organization presenting these things. This is about getting a group of organizations to work together. Every single organization that’s in that room has branding rules and logos. . . . We’ve worked hard to rebrand ourselves as the coalition. — Communication lead

You have to be okay in your communication efforts knowing that sometimes when the partner is the carrier of the message, that as long as the community is becoming aware, you’re doing your job. Even if your name’s not the final one on it, the goal is that community awareness increases. — Communication lead

## Theme 3: Channels

According to programs, operating in a less populous area has advantages for mass communication. Programs felt it was relatively easy to access existing networks for communication activities in nonmetropolitan regions using existing networks to involve partners and access channels for mass communication. In small, close-knit communities, these relationships were frequently useful and reliable avenues for message dissemination.

Special considerations apply to the selection of communication channels in nonmetropolitan regions. Programs serving rural areas noted that channels designed to maximize reach in larger markets (eg, television or outdoor advertising) need to be carefully considered in less populous regions. For example, billboards lack exposure if placed in areas without heavy traffic flow, transit advertising is not relevant in areas with limited to no public transportation, and television and radio broadcasts may reach beyond the key population or geographic region, or alternately, may not reach isolated areas. Programs frequently used alternative, often economical, channels to communicate to these audiences, including social media posts and paid messaging on social media platforms, local-access or cable-access television or radio, internet radio programs, podcasts, newsletters, and mass mailings. Almost all refined and expanded their social media presence during the funding period. Programs found opportunities to promote their activities by using earned media through local publications and news stories. Programs also observed that earned media coverage generated interest and encouraged the replication of program activities by other small communities or neighboring regions.

### Quotes regarding channels

#### Programs reported relatively easy access to existing networks for communication activities in nonmetropolitan regions.

Most people within the health care arena are aware that we’re here. [It] is maybe easier than some of the bigger markets where there are just so many layers of . . . providers. You just know each other here. — Communication lead

I haven’t run into a situation where someone has said no. That’s [attributable to] size. Even in large cities and large communities, there are niches and there are smaller communities. . . . There is just an ease [in these clusters]. — Communication lead

#### Special considerations applied to the selection of communication channels in nonmetropolitan regions.

How we handle communications within [our city] is different than how we handle it within a rural community. [In a city of 80,000], that’s going to our radio stations or our TV outlets. . . . Now that we’ve reached out to the smaller communities, we’ve had to learn the ropes for small towns. — Program manager

The thing we started doing this year, as well, is Facebook paid messaging. . . . What I really liked about the Facebook option, when you do a newspaper ad, you can’t say “I only want to reach 18 to 25 [year old] African Americans that live in this zip code.” It ends up [being] whoever picks up the newspaper, which sometimes is our population and sometimes it’s not. [Facebook] allowed me to really tailor down. — Communication lead

Radio, in particular, is effective. I know, having lived in the city in the past, it’s not a target or a way to reach everyone, but here, with a few stations you can really reach a huge number of people by getting on a few key radio programs of that sort of thing. — Program manager

When we look at a limited budget [next year], our focus will definitely be on more social media because that’s what we’re hearing constantly. . . . It’s really realizing and accessing your audience exactly where they are. Had all projects in the past had someone in communications, that would have continually been the focus. — Program manager

One of the foundations of our [communication] plan that I think has been most successful is the social media work. — Program manager

#### Earned media coverage encouraged replication of program activities in other small communities or neighboring regions.

It seems like when there’s a rural community that’s doing something, there’s another community that hears about it. Then they may want to do it because the other community did it. By having the media pick up on it and say, “Hey, this is what small town ABC is doing,” I think that has helped persuade other small towns to decide [to do it, too]. Then they’re more willing to participate in some of our initiatives. — Program manager

## Theme 4: Cultural Competence

REACH programs emphasized the importance of allowing cultural contexts to guide program planning, implementation, and mass communication when addressing health disparities. Celebrating and embracing the cultural roots of the community can empower and encourage community members in the process of communicating about public health programs. According to interviewees, consideration of cultural and spiritual aspects like language, ethnic background, church events, and cultural practices reinforced powerful community connections and improved message dissemination. Programs valued the involvement of community members in the cocreation and testing of messaging, particularly when program staff were not from the same social and cultural backgrounds as audiences. The tribal programs emphasized the importance of considering the unique attributes and infrastructure of tribal communities in all aspects of program planning and implementation, including approaches to mass communication.

### Quotes regarding cultural competence

#### Celebrating the cultural roots of the community empowered and encouraged community members in the process of communicating about public health programs.

I think the other reason why this has been so successful is the [production] company we chose to work with made a huge difference. . . . They’re Native. . . . Before I got into this, I knew if we did our media right, we could impact a lot of people nationwide in Indian Country. I do wellness and healing all across Indian Country. I know the importance of striking that emotion. — Program manager

You have to look at the heart of the people you’re working with. You have to connect at where they’re at. If the majority of your people are Latino then you have to connect with their culture. If the majority of your people are Black, then you have to connect with their culture. That’s what I think is our biggest takeaway from this. — Program manager

We tried to get into the community as deeply as we could. That really guided a lot of what we did, especially because we realized how much culture was playing into the mix. . . . I think it really came down to the nuances between those 2 cultures [and that] changed the way we did everything. . . . What we realized is, we needed to go to places that were either designed to be culturally specific or naturally became culturally specific locations. We could really tailor the intervention to each of the communities a little better. — Communication lead

People were shocked to see a billboard in Spanish, but there’s the benefit of shock value and catching someone’s eye and realizing that message is solely for them. . . . Even now there’s been commercials in Spanish on English-[language] TV stations, because it communicates, “We know you’re watching too.” It’s a respect value and having the [platform] to say, “We know you’re [in] the minority, but we care enough to ensure this message reaches you.” — Communication lead

## Discussion

Small- to mid-sized public health programs may be unsure of the feasibility of using mass communication in their program activities. REACH/PICH programs in nonmetropolitan regions demonstrated the successful use of mass communication strategies to promote and support programmatic efforts. The experiences suggest that nonmetropolitan communities can achieve communication objectives through various adaptive approaches, frequently leveraging local partners, networks, and resources.

Undertaking mass communication for public health programs requires defined objectives, understanding the audiences to reach and the best channels and strategies to reach them, and evaluation methods to track changes over time — each grounded in health behavior change and communication theory (7). To implement mass communication activities successfully, programs can hire dedicated communication staff and external media contractors with public health experience and use a mix of cost-effective media channels and categories to reach key audiences.

Audience research using established methodologies — such as focus groups, in-depth interviews, or surveys — supported the creation of mass communication approaches that reflected the local contexts of the communities in which the programs operated. Culture has been identified as an influential factor in how message content, structure, sources, and channels are perceived and received by key audiences, and is associated with multiple facets of health behavior (8). Health programs can look beyond epidemiological and demographic characteristics (eg, age, race/ethnicity, geographic boundaries) to consider culture when understanding key audiences (8). Incorporating cultural aspects into mass communication efforts can increase the salience of health messages and programs offered in communities.

These examples illustrate that it is feasible for public health programs in nonmetropolitan regions to increase internal capacity for mass communication to reach target audiences. CDC provides resources on health communication and social marketing for public health programs that are applicable in nonmetropolitan settings (9). These program experiences may inform future research and evaluation to identify the most effective communication strategies in these settings. With dedicated technical assistance and resources, programs serving small to mid-sized populations can develop communication activities that increase internal capacity and gain organizational support for communication, establishing the groundwork for sustaining communication efforts.
